# Massively Dilated Lower Pole Ectopic Megaureter with Involuted Lower Pole Renal Moiety and Collecting System: An Exception to the Meyer-Weigert Rule

**DOI:** 10.7759/cureus.7577

**Published:** 2020-04-07

**Authors:** Gavin Stormont, John Makari, Angela Beavers, Claudia Berrondo

**Affiliations:** 1 Urology, University of Nebraska Medical Center, Omaha, USA; 2 Urology/Pediatric Urology, Children's Hospital and Medical Center, Omaha, USA; 3 Radiology/Pediatric Radiology, Children's Hospital and Medical Center, Omaha, USA; 4 Surgery/Pediatric Urology, University of Nebraska Medical Center, Omaha, USA; 5 Pediatric Urology, Children’s Hospital and Medical Center, Omaha, USA

**Keywords:** duplicated collecting system, meyer-weigert rule, megaureter, ectopic ureter

## Abstract

Duplicated collecting systems have a predictable draining pattern that is described by the Meyer-Weigert rule. When there are abnormalities associated with duplicating collecting systems, the upper pole moiety drains inferomedially (most commonly associated with obstruction), and the lower pole moiety drains superolaterally (most commonly associated with vesicoureteral reflux). We present a case of an infant with a duplicated collecting system that violates the Meyer-Weigert rule with lower pole megaureter with massive dilation, ectopic insertion, and associated involuted lower pole renal moiety. To our knowledge, this is the only reported case of a lower pole ectopic ureter with an involuted lower pole renal moiety.

## Introduction

Duplication of the renal collecting system is one of the most common congenital urologic abnormalities. In cases of complete ureteral duplication, the drainage of the ureters is predicted by the Meyer-Weigert rule in which the upper pole ureter drains inferomedially and the lower pole ureter drains superolaterally [1,2]. Most cases of renal duplication are not associated with other abnormalities. However, when abnormalities are present, the upper pole ureter is typically associated with obstruction from ectopic insertion, and the lower pole ureter is typically associated with vesicoureteral reflux [3]. We report a rare case of an exception to the Meyer-Weigert rule in which there is lower pole obstruction secondary to ectopic insertion with involution of the lower pole renal moiety.

## Case presentation

**Figure 1:**
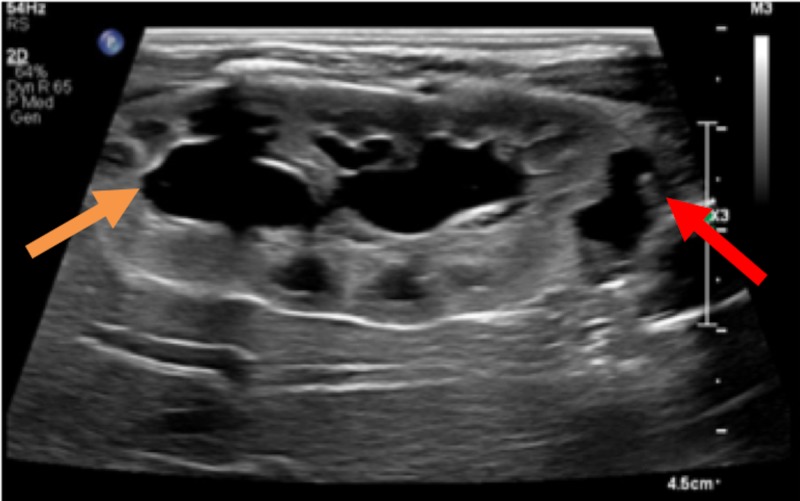
Renal ultrasound Prone ultrasound image of the right kidney in the sagittal plane demonstrates a duplicated collecting system. The orange arrow points to the upper pole collecting system and the red arrow points to the lower pole collecting system.

A seven-day-old male presented to pediatric urology with antenatally diagnosed right hydronephrosis with a severely dilated ureter. A follow-up ultrasound one month later demonstrated increased ureteral dilation which was further evaluated with a voiding cystourethrogram which was normal, and a renogram which demonstrated approximately equal differential function with obstruction. He was initially managed with serial renal ultrasound and observation. Follow-up ultrasound at six months of age demonstrated concern for possible duplication of the right collecting system (Figure 1).

**Figure 2:**
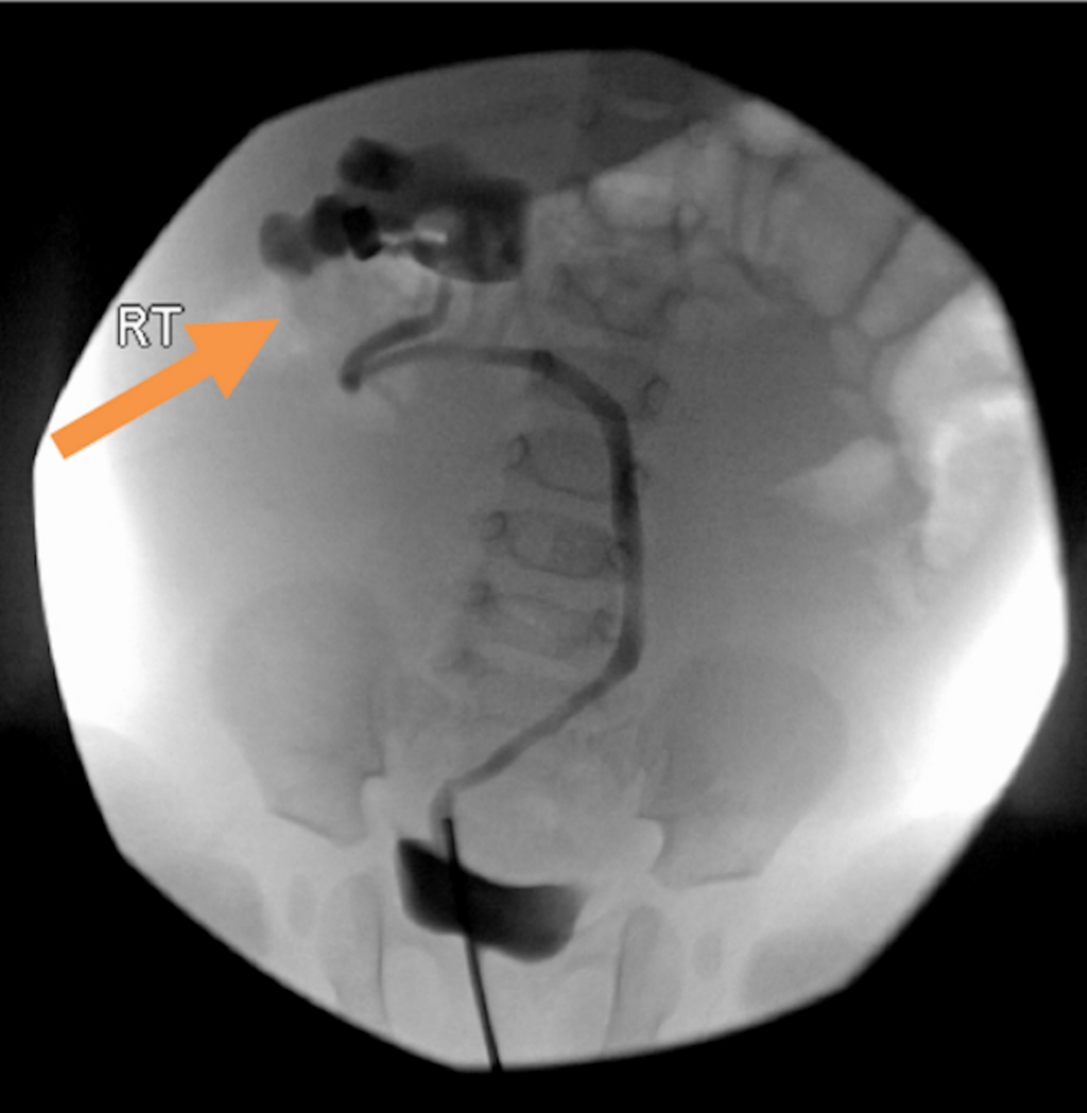
Retrograde pyelogram Retrograde pyelogram of the upper pole non-dilated but deviated ureter and abnormal orientation of the right upper pole collecting system. The orange arrow points to the upper pole collecting system.

Cystoscopy at eight months of age revealed a single right and left ureteral orifice orthotopically located. Left retrograde pyelogram was normal. Right retrograde pyelogram revealed a non-dilated but deviated right upper pole ureter (Figure 2). The right lower pole ureteral orifice was not identified.

**Figure 3:**
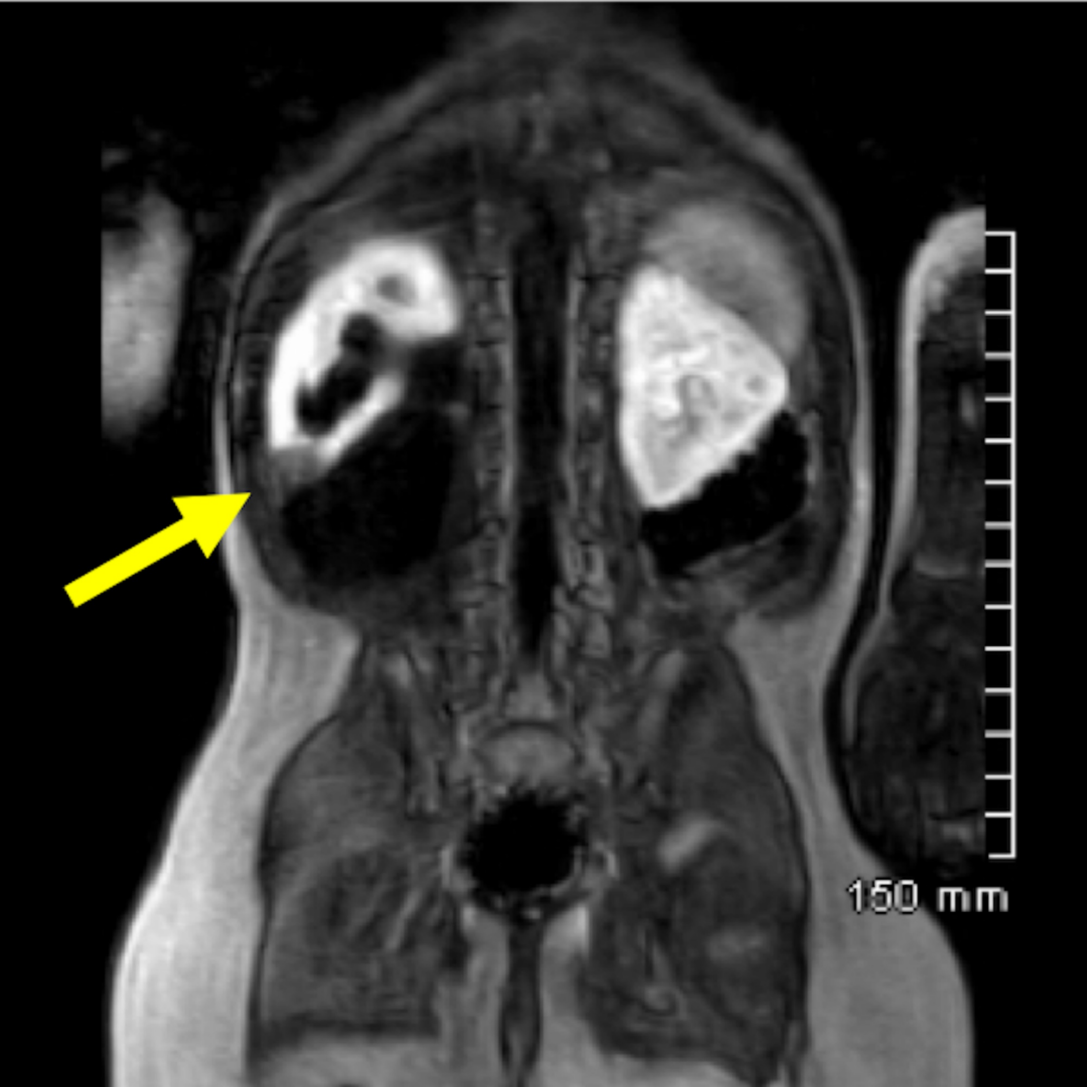
Magnetic resonance urogram Dynamic T1-weighted post contrast coronal image of magnetic resonance urogram. The yellow arrow shows scarring in the lower pole of the right kidney.

**Figure 4:**
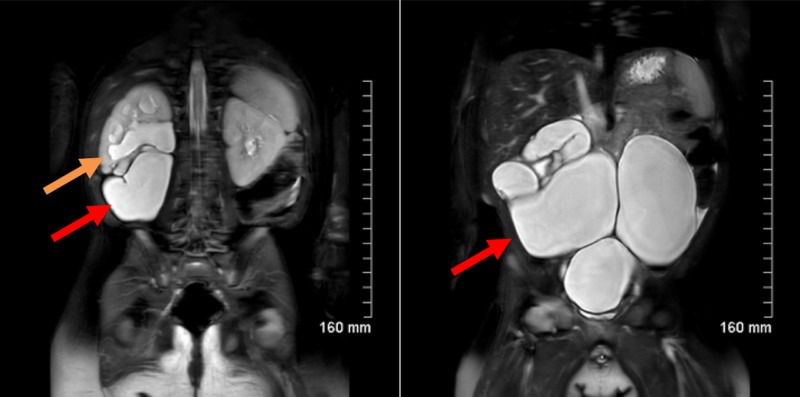
Magnetic resonance urogram Magnetic resonance urogram showing a dilated lower pole ureter and upper pole hydronephrosis. The orange arrow points to the upper pole collecting system, and the red arrow points to the massively dilated lower pole ureter.

Magnetic resonance urography confirmed a duplicated collecting system on the right with upper pole hydronephrosis and a tortuous ureter due to displacement by the massively dilated lower pole ectopic ureter and scarring of the lower pole renal moiety (Figures 3, 4). 

**Figure 5:**
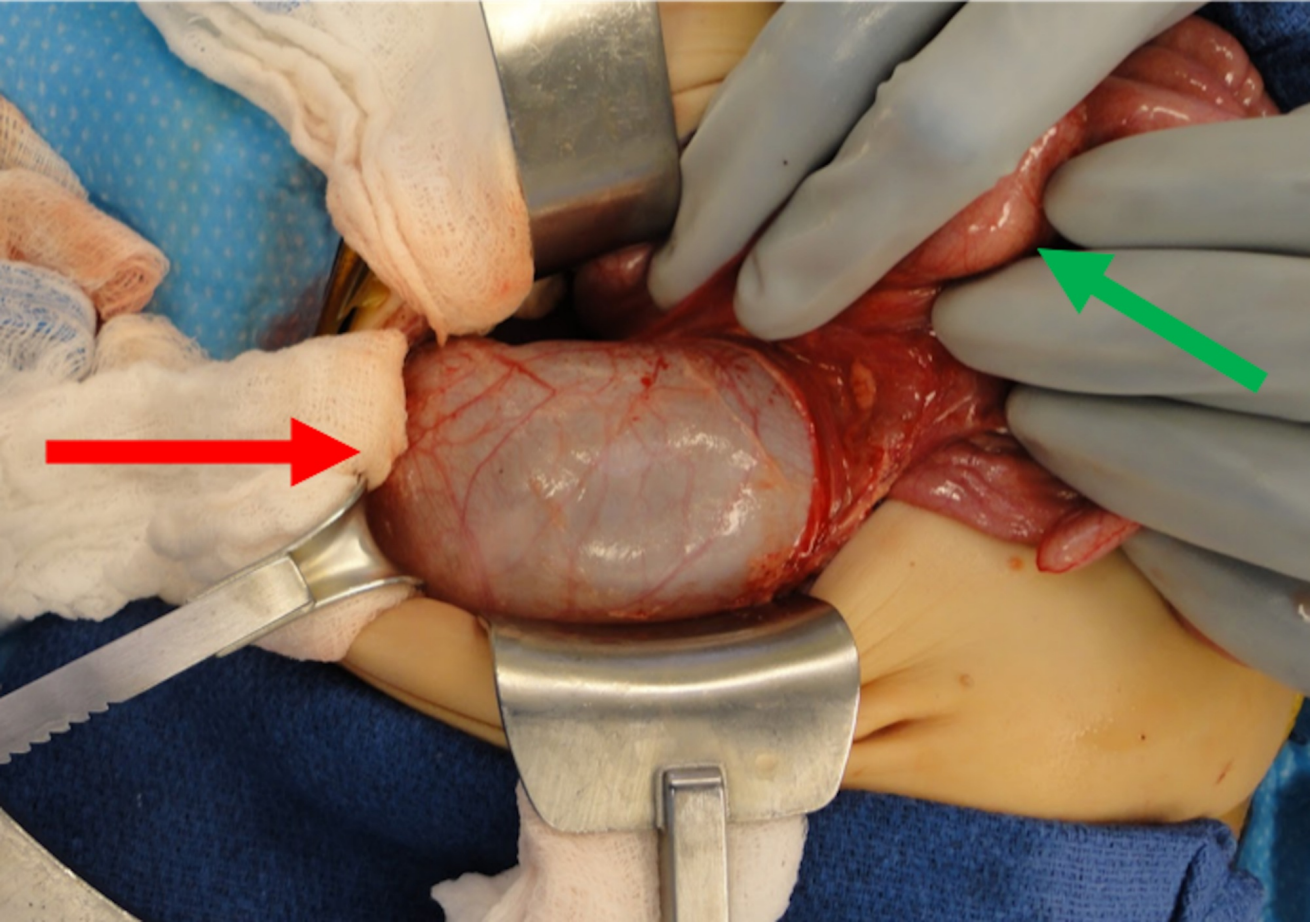
Intraoperative photo Intraoperative photo of the dilated right lower pole ureter and displaced small bowel. The red arrow points to the massively dilated lower pole ureter. The green arrow points to the displaced small intestine.

He had normal renal function with a creatinine of <0.15 mg/dl. Due to the space-occupying nature of the right lower pole ureter, surgical excision with right lower pole heminephrectomy with ureterectomy was pursued. Intraoperatively, the right lower pole ureter was massively dilated causing displacement of the upper pole ureter, small bowel, and ascending colon (Figure 5).

**Figure 6:**
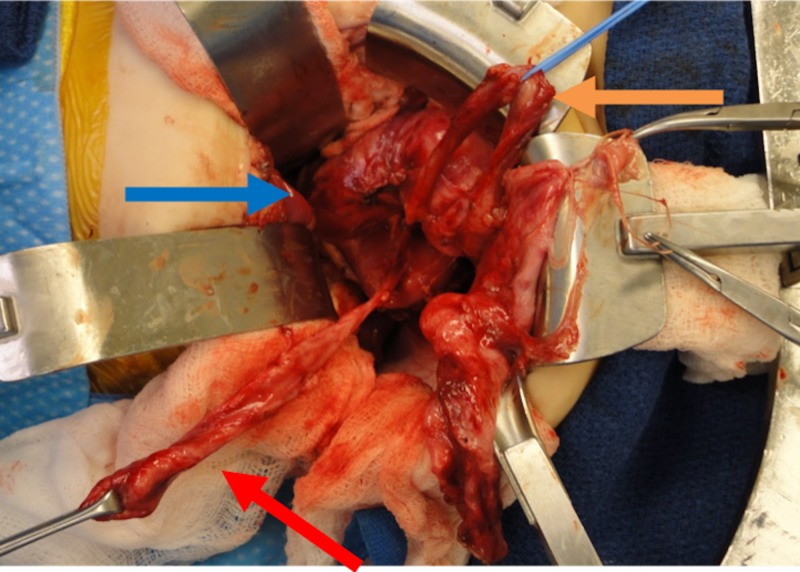
Intraoperative photo Intraoperative photo of the right upper pole renal moiety with associated ureter and lower pole ureter originating from an involuted lower pole renal moiety. The blue arrow points to the upper pole renal moiety, the orange arrow points to the upper pole ureter, and the red arrow points to the lower pole ureter.

There was visible lower pole moiety renal parenchymal tissue or dilated collecting system associated with the ureter, so the ureter was ligated where it appeared to arise from the normal appearing renal parenchyma of the upper pole moiety (Figure 6). The proximal segment of the ureter was excised down to the level of the iliac vessels. The distal segment of the ureter was marsupialized. The ectopic insertion of the lower pole ureter was unable to be visualized. 

His postoperative course was uneventful, and he was discharged home on postoperative day 2. Pathology revealed ureteral tissue with hypertrophy of the muscularis, fibrosis, and patchy mild chronic inflammation consistent with a duplicated blind ending ureter. 

## Discussion

Ureteral duplication is one of the most common congenital anomalies, and the drainage pattern of completely duplicated ureters is predicted by the Meyer-Weigert rule [1,2]. This is an unusual case of a duplicated collecting system with lower pole obstructed and ectopic megaureter with an involuted lower pole renal moiety. Although several previous case reports demonstrating exceptions to the Meyer-Weigert rule have been previously published as described below, to our knowledge this is the only case with associated involution of the lower pole renal moiety. 

Jain et al. reported a similar case of a 10-year-old boy with a duplicated collecting system with dysplastic lower pole moiety and ectopic ureter [4]. Darr et al. described a similar case of a duplicated collecting system with lower pole megaureter without inferolateral insertion of the lower pole ureter into the bladder, and with associated dysplastic lower pole renal moiety [5]. Brown et al., Mishra and Elliott, and Slaughenhoupt et al. each reported cases of lower pole ectopia to a Wolffian duct structure [6-8]. In each of these cases, the ectopic ureter appeared to arise from a lower pole calyx. Ahmed described a case of exception to the Meyer-Weigert rule of a three-year-old female with a duplicated collecting system with superolateral insertion of an upper pole ureter with upper pole renal moiety involution and lower pole megaureter with inferomedial ureteral insertion with associated ureterocele [9]. 

These cases all demonstrate rare exceptions to the Meyer-Weigert rule, all with varying anatomy. Currently, there is no embryologic explanation for the development of these anatomic anomalies, and reports in the literature are rare.

## Conclusions

In our case, we demonstrate a rare scenario of an orthotopic upper pole ureter draining a normal upper pole renal moiety with an ectopic and obstructed lower pole ureter with involuted lower pole renal moiety. Lower pole ureteral obstruction is rare with few cases reported in the literature. These cases serve as a reminder to complete full anatomic evaluation with radiographic imaging in order to plan the appropriate surgical intervention. 
